# Dietary supplements for prediabetes: A protocol for a systematic review and meta-analysis

**DOI:** 10.1097/MD.0000000000020347

**Published:** 2020-05-15

**Authors:** Dongying Liu, Qing Wen, Min Liu, Yang Gao, Lihong Luo, Zhuo Zhang, Qiu Chen

**Affiliations:** Hospital of Chengdu University of Traditional Chinese Medicine, Chengdu, Sichuan, China.

**Keywords:** diabetes prevention, dietary supplements, prediabetes, protocol, systematic review

## Abstract

**Background:**

The number of prediabetic individuals is at a high level worldwide and they have an increased risk of developing diabetes, causing severe physical impairment and heavy financial burden. Recently, using various dietary supplements is increasingly common, and relevant trials of different diseases are increasing correspondingly. The effects of dietary supplements have been confirmed in some studies among prediabetic individuals. However, there remains no comprehensive systematic review to assess the efficacy and safety of dietary supplements intake in prediabetic individuals.

**Methods:**

We plan to search and retrieve applicable randomized controlled trials of dietary supplements for prediabetic individuals in the following databases before June 2020: PubMed, Web of Science, EMBASE, the Cochrane Library, the Cochrane Central Register of Controlled Trials(CENTRAL), Allied and Complementary Medicine Database(AMED), Chinese Biomedical Literature database, Wan Fang database, Chinese Scientific Journal database (VIP), Chinese National Knowledge Infrastructure database(CNKI), and the ClinicalTrials.gov website. Two reviewers will separately perform study selection, data extraction, methodological quality assessment and quality of evidence assessment. Data analysis and publication bias will be conducted by Review Manager 5.3.

**Results and conclusions:**

This evidence-based medicine systematic review will prove the efficacy and safety of multifarious dietary supplements for prediabetes.

**Ethics and dissemination:**

As this systematic review is based merely on already published literature, no approval of the ethics committee is required. We will disseminate this systematic review to a peer-reviewed journal.

**Protocol registration number:**

INPLASY202040057.

## Abstract 2

Supplemental Digital Content is available in the text

## 1 Introduction

Prediabetes is recommended to be named intermediate hyperglycemia by the World Health Organization(WHO) which represents the metabolic state between normal glucose tolerance and diabetes.^[1]^ There remain different types and diagnostic criteria of prediabetes:

1. impaired fasting glucose(IFG): fasting plasma glucose 6.1 to 6.9 mmol/L(the WHO criteria) or 5.6 to 6.9 mmol/L(the American Diabetes Association(ADA) guideline),
2. impaired glucose tolerance(IGT): 2-hour plasma glucose 7.8 to 11.0 mmol/L(both WHO and ADA),
3. raised hemoglobin A1c(HbA1c): 39 to 47 mmol/mol(5.7–6.4%) defined by ADA or 42 to 46 mmol/mol(6.0–6.4%) defined by the International Expert Committee(IEC),
4. the coexistence of IGT and IFG.^[2,3,4]^

According to the International Diabetes Federation, the number of adults with impaired glucose tolerance or diabetes was 318 million and 415 million in 2015, respectively, and it is estimated that the number will rise to 482 million and 642 million in 2040.^[5,6]^ Compared with normoglycemic individuals, people with prediabetes are related to a higher rate of coronary heart disease, composite cardiovascular disease, stroke, chronic kidney disease, and all-cause mortality.^[7,8]^ Among adults aged 20 to 79 years old in 2019, there were about 4.2 million deaths attributable to diabetes with its various complications. Direct health expenditure of diabetes has reached USD 760 billion, let alone the impact on the labor market caused by some special circumstances of diabetes like disability.^[9,10]^ Although approximately 5% to 10% of prediabetic individuals develop type 2 diabetes mellitus(T2DM) every year and 70% eventually reach the irreversible diabetic endpoint, some of them do not convert to diabetes and finally return to normoglycemia.^[11]^ Hence, taking effective measures before developing diabetes seems essential.

Abnormity of insulin sensitivity and β-cell function which thought to be the main physiopathologic mechanism exists many years before diabetes development. Carrying out interventions to delay or prevent β-cell failure seems to be indispensable in diabetes prevention.^[12]^ Lifestyle modification(LM), with minimal risk of side effects, is the cornerstone for diabetes prevention which includes increased exercise, healthy diet, and weight reduction. However, it is not easy to follow continuous intervention and rigorous compliance in the long run, which make LM difficult to perform optimally.^[13,14]^ Many articles have reported that pharmacological interventions including metformin, pioglitazone, acarbose, liraglutide, orlistat, etc. can be used to prevent diabetes, mitigate some of the potential consequences of progressing to diabetes, and prevent underlying harms of prediabetes itself.^[11,15]^ However, none of them are recommended by the U.S. Food and Drug Administration(FDA) other than metformin which has a validated evidence base and long-term safety, especially for those high-risk individuals with body mass index(BMI) ≥35 kg/m^2^.^[16]^

As an important form of complementary and alternative medicine(CAM), dietary supplements(DS) is defined as a product made-up of herbs or other botanicals, minerals, vitamins, enzymes, amino acid, or dietary substances. DS, which appears on the market as tablets, capsules, softgels, gelcaps, powders, and liquids, intends to supplement meals by increasing overall dietary intake, or an extract, constituent, metabolite, concentrate, or combination of any before-mentioned ingredients.^[17]^ To maintain overall general health, prevent disease, compensate for unhealthy lifestyles or treat specific diseases and conditions, more people tend to take DS which has a stepwise increased rate of using. During a 30-day period in the US, half of the adults (52%) take at least one DS, and multivitamin-mineral products are used most commonly.^[18,19]^ Among people with prediabetes, dissatisfaction with conventional medicines, and pursuit of naturopathy often promote individuals taking nontraditional alternatives, such as DS.

There have been some systematic reviews of diabetes management with dietary supplements, providing limited evidence on the effects of coccinia indica, trigonella foenum, American ginseng, aloe vera, chromium, vanadium, ginger, curcuminoid and cinnamon supplements for insulin resistance, glycaemic and lipid control.^[20,21]^ Some of these supplements are still useful in prediabetic individuals. Plant sterols and omega-3 fatty acids were supposed to be useful in preventing IGR progressing to diabetes because of the improvement of glucose metabolism, lipid metabolism, insulin resistance and inflammation.^[22]^ A study about zinc supplementation explored the benefits of reducing blood glucose levels, insulin resistance while improving β-cell function and even lowering the prevalence of T2DM.^[23]^ In addition, unlike prescription medicine, DS is easy to obtain in grocery stores, pharmacies and supermarkets, and plenty of individuals supplement DS themselves without detailed knowledge and any suggestion of medical practitioners. Hence, the efficacy and safety of DS need more attention.^[24,25]^ However, no comprehensive systematic reviews remain so far, and we aim to collate the evidence on DS for prediabetes to evaluate its efficacy and safety, so as to provide an overall assessment and serve as a reference for consumers, practitioners and future researchers in this field.

## 2 Methods

### 2.1 Study design and registration

We have registered this systematic review on the International Platform of Registered Systematic Review and Meta-analysis Protocols as INPLASY202040057(https://inplasy.com/). This protocol was written and reported under the Preferred Reporting Items for Systematic Review and Meta-Analysis Protocols (PRISMA-P) standards.^[26]^ And we are going to perform and report this meta-analysis following the Preferred Reporting Items for Systematic Reviews and Meta-Analysis(PRISMA).^[27]^

### 2.2 Eligibility criteria

#### 2.2.1 Types of studies

Only randomized controlled trials (RCTs) of dietary supplements for prediabetes irrespective of language and publication status will be included in this review to ensure high-quality evidence. Those animal experiments, cohort, case-control, case series, and observational studies are out of our choice as well as duplicated publications and study protocols.

#### 2.2.2 Types of participants

We intend to incorporate participants with prediabetes diagnosed as IGT, IFG, raised HbA1c, or status of IGT and IFG coexistence irrespective of age, gender and ethnicity. Participants involved in eligible trials will not be included in the design and implementation of this systematic review.

#### 2.2.3 Types of interventions and controls

RCTs of dietary supplements as the intervention in the experimental group compared with placebo, conventional treatment (such as LM and medicine) or blank control will be included in our study. We plan to exclude trials of traditional Chinese herbal formulas, Chinese patent medicines and vitamin D as they have been studied previously.^[28,29,30]^

#### 2.2.4 Types of outcomes

The incidence of diabetes and the rate of normoglycemia will be reported as primary outcomes in this review. Secondary outcomes will be fasting blood glucose (FBG), postprandial blood glucose (PBG), glycosylated hemoglobin A1c (HbA1c), fasting insulin (FINS), insulin sensitivity, adverse events, plasma lipids, inflammatory markers, and body mass index (BMI).

### 2.3 Search strategy

#### 2.3.1 Electronic searches

Two reviewers will independently search the following electronic databases to identify eligible trials before June 2020: PubMed, Web of Science, EMBASE, the Cochrane Library, the Cochrane Central Register of Controlled Trials(CENTRAL), Allied and Complementary Medicine Database(AMED), Chinese Biomedical Literature database, Wan Fang database, Chinese Scientific Journal database (VIP), Chinese National Knowledge Infrastructure database(CNKI), and the ClinicalTrials.gov website. The retrieval strategy will be developed and completed by all the researchers combining subject words and free words. We will use the following terms: dietary supplement^∗^, food supplement^∗^, herbal supplement^∗^, nutraceutical^∗^, nutriceutical^∗^, neutraceutical^∗^, supplement^∗^, vitamin^∗^, herb^∗^, protein, mineral, enzyme^∗^, amino acid, prediabetes, prediabetic, pre-diabetic, pre-diabetes, impaired fasting glucose, impaired glucose tolerance, impaired glucose regulation, IFG, IGR, IGT, diabetes prevention, hyperglycemia, randomized controlled trial, RCT, controlled clinical trial, randomized, trial, random, placebo, groups. Details of the search strategy of the PubMed database are presented in supplementary file 1.

#### 2.3.2 Other resources

Bibliography lists of included trials, relevant systematic reviews and meta-analyses will be searched manually to identify additional eligible studies.

### 2.4 Data collection and analysis

#### 2.4.1 Selection of studies

**Figure 1:**
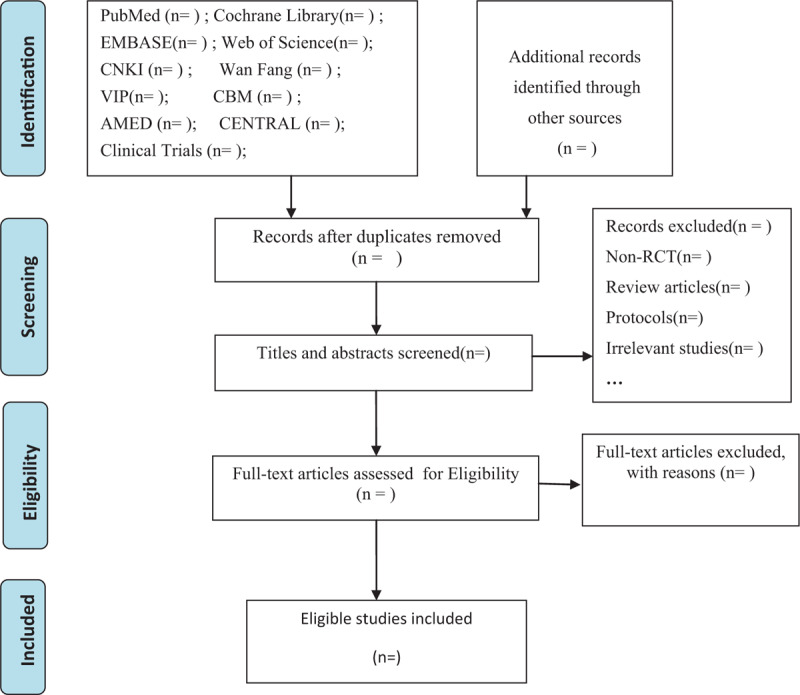
Flow chart of the study selection process. Arrows = flow directions or reasons for exclusion.

The selection process for eligible trials will be implemented by 2 independent researchers. We plan to import all the retrieved articles to the document management software EndnoteX9 (Thomson Research Soft, Stanford, Connecticut) and initially sift titles and abstracts of records, selecting appropriate RCTs. Then full texts of the remaining literature will be downloaded for further screening to retain trials meeting the inclusion criteria. Any discrepancy will be resolved by discussing or seeking advice from the third party. The flow chart here will present the detailed screening process showing the number of included or excluded papers and reasons at each stage to ensure transparency (Fig. 1).

#### 2.4.2 Data extraction and management

A data extraction template predesigned by all the review authors will be used by 2 independent researchers simultaneously for data extraction. Consult to a third reviewer if any inconsistency appears in careful calibration of detailed information extracted respectively to resolve the doubts and make the information comprehensive and precise. When we encounter insufficient or unclear data in the involved study, we will contact the authors by email or phone to acquire further information. We will extract the following items:

1. Publication information: title, author, publication year, source;
2. Study characteristics: design, sample size, duration of follow-up, randomization methodology, allocation concealment, blinding;
3. Participants: gender, age, number of each group, types of prediabetes and its definition criteria;
4. Interventions: types of interventions, types of controls, dosage form, dose, duration of intervention, route of administration;
5. Outcomes: outcome indicators, adverse events, number of withdrawals, and participants completing trials, the reason for withdrawing.

#### 2.4.3 Missing data management

If we fail to obtain sufficient data by contacting the authors, available case analysis or sensitivity analysis will be performed, proving, and discussing whether missing information potentially affects the overall results.

#### 2.4.4 Risk of bias assessment

The quality of methodology will be appraised independently by 2 researchers based on the Cochrane collaborations risk of bias tool in 7 dimensions: random sequence generation, allocation concealment, blinding of participants and personnel, blinding of outcome assessment, incomplete outcome data, selective outcome reporting, and other biases.^[31]^ The risk of bias of each item will be categorized as “low”, “uncertain” or “high”, and discrepancies are going to be settled by discussion or consultation with another review author.

#### 2.4.5 Data synthesis and statistical analysis

We propose to conduct this meta-analysis by using the Review Manager 5.3 statistical software. We will analyze all the results with 95% confidence intervals (CIs). To evaluate the treatment efficacy, the dichotomous variables will be merged to risk ratio (RR). For continuous variables, we plan to estimate the overall results with mean difference (MD) or standard mean difference (SMD), which mainly depends on the consistency or inconsistency of the outcome scale.

For the same outcome measure, where there remain 2 or more studies with the same type of intervention and the clinical heterogeneity is not considered by the reviewers, we intend to conduct a quantitative synthesis. Considering studies with different types of interventions are able to meet this condition, we will analyze by groups which are divided according to different types of outcomes and interventions. In terms of assessing and quantifying the heterogeneity of outcomes, the Chi-Squared test and the *I*-square (*I*^2^) statistic will be performed. If the *P-*value < .10 in the Chi-Squared test, it reveals the existence of heterogeneity. If *I*^2^ < 50%, we will select the fixed effects model to estimate the aggregated effects given the absence of apparent heterogeneity. On the contrary, the random effects model will be utilized to pool the results together when 50% ≤ *I*^2^ < 75%, and moderate heterogeneity is taken into consideration. Where *I*^2^ ≥ 75%, we will give up pooling the data to perform narrative analysis given the presence of substantial heterogeneity. Besides, qualitative description will also be conducted to summarize and explain information and discoveries of the study where no more than 1 trial with the same intervention exists.

#### 2.4.6 Subgroup analysis

This meta-analysis will be performed based on subgroups of different types of dietary supplements. At present, we do not preplan other subgroup analyses. If heterogeneity exists, methodological and clinical differences between involved trials will be considered first. The analyses of other influence factors (types of prediabetes, duration of treatment, and dose) will be determined on a case-by-case basis.

#### 2.4.7 Publication bias

If there remain at least 10 trials reporting the same comparison, we will employ a funnel plot to detect potential reporting bias. In terms of complex explanations of a funnel plots asymmetry, the results of different situations will be interpreted cautiously.

#### 2.4.8 Sensitivity analysis

To test the robustness and reliability of each outcome, the possible sources of heterogeneity, sensitivity analysis will be performed. We plan to omit studies with high risk of bias or insufficient data, and the meta-analysis afterward will be performed repeatedly.

#### 2.4.9 Quality of evidence

The Grading of Recommendations Assessment, Development and Evaluation (GRADE) method is applicable for this systematic review to assess the quality of evidence.^[32]^ We will rate the evidence as “high”, “moderate”, “low”, or “very low” in a conclusive table using the GRADE profiler 3.2.

## 3 Discussion

The number of prediabetic individuals is at a high level worldwide and they have an increased risk of developing T2DM which eventually causes severe physical impairment and heavy financial burden.^[9,10]^ LM recommended as a basic method of intervention for prediabetes needs long-term continuous intervention and rigorous compliance so that it is hard to persist.^[14]^ For drugs, none of them has been recommended as part of routine clinical care, and their usage requires comprehensive consideration of patient condition, durable efficacy and side effects.^[16]^ DS, a common type of complementary and alternative medicine, is universally and more frequently used recently, and no exception in the prediabetic people. There has been some relevant evidence to prove its effectiveness. In view of the complexity of its components and the easy accessibility, DS needs more scientific evidence and stronger regulation from the FDA.^[25]^ This systematic review aims to provide a summary of evidence concerning multiple dietary supplements used in prediabetic individuals to prevent or delay diabetes, control the metabolic status and confirm the safety. Furthermore, we may as well offer suggestions of effective and appropriate options based on the analysis, benefiting for consumers, practitioners and future researchers. However, some possible limitations may occur in this study. This review will not include trials with the intervention of DS plus another therapy, and we can not rank each DS to determine the optimal supplementation due to the limitations of the methodological approach. According to our tentative retrieval, the number of studies of some types of DS seems to be relatively small. In addition, high clinical and statistical heterogeneity may arise from different types of prediabetes, duration of treatment, dosage, dosage form, gender, or age.

## Author contributions

**Conceptualization:** Dongying Liu.

**Data curation:** Qing Wen, Lihong Luo, Zhuo Zhang.

**Formal analysis:** Dongying Liu, Qing Wen.

**Methodology:** Dongying Liu.

**Project administration:** Dongying Liu, Qiu Chen.

**Resources:** Dongying Liu, Qiu Chen.

**Software:** Dongying Liu, Qiu Chen.

**Supervision:** Qiu Chen.

**Writing – original draft:** Dongying Liu.

**Writing – review & editing:** Min Liu, Yang Gao.

## Supplementary Material

Supplemental Digital Content
