# Supplementary material for: Dietary supplements for prediabetes: A protocol for a systematic review and meta-analysis
Source: Medicine (Baltimore). 2020 May 15;99(20):e20347. doi: 10.1097/MD.0000000000020347 (PMC7253655; doi:10.1097/MD.0000000000020347)
Supplement: Supplemental Digital Content [file medi-99-e20347-s001.doc]

**Dietary Supplements for Prediabetes: Protocol for a Systematic Review and Meta-analysis**

**Search strategy used in PubMed database:**

#1 dietary supplement*[Title/Abstract]

#2 food supplement*[Title/Abstract]

#3 herbal supplement*[Title/Abstract]

#4 nutraceutical*[Title/Abstract]

#5 nutriceutical*[Title/Abstract]

#6 neutraceutical*[Title/Abstract]

#7 nutrient*[Title/Abstract]

#8 nutrition[Title/Abstract]

#9 supplement*[Title/Abstract]

#10 herb*[Title/Abstract]

#11 vitamin*[Title/Abstract]

#12 protein[Title/Abstract]

#13 enzyme*[Title/Abstract]

#14 mineral[Title/Abstract]

#15 amino acid[Title/Abstract]

#16 extract*[Title/Abstract]

#17 #1 OR #2 OR #3 OR #4 OR #5 OR #6 OR #7 OR #8 OR #9 OR #10 OR #11 OR #12 OR #13 OR #14 OR #15 OR #16

#18 prediabetes[Title/Abstract]

#19 prediabetic *[Title/Abstract]

#20 pre-diabetes[Title/Abstract]

#21 pre-diabetic[Title/Abstract]

#22 impaired fasting glucose[Title/Abstract]

#23 IFG[Title/Abstract]

#24 impaired glucose tolerance[Title/Abstract]

#25 IGT[Title/Abstract]

#26 impaired glucose regulation[Title/Abstract]

#27 IGR[Title/Abstract]

#28 hyperglycemia[Title/Abstract]

#29 diabetes[Title/Abstract]

#30 prevent*[Title/Abstract]

#31 #29 AND #30

#32 #18 OR #19 OR #20 OR #21 OR #22 OR #23 OR #26 OR #27 OR #28 OR #31

#33 randomized controlled trial[Publication Type]

#34 controlled clinical trial[Publication Type]

#35 randomized[Title/Abstract]

#36 controlled[Title/Abstract]

#37 trial[Title/Abstract]

#38 random[Title/Abstract]

#39 placebo[Title/Abstract]

#40 groups[Title/Abstract]

#41 #33 OR #34 OR #35 OR #36 OR #37 OR #38 OR #39 OR #40

#42 #17 AND #32 AND #41
